# Supplementary material for: Consensus on maturity-related injury risks and prevention in youth soccer: A Delphi study
Source: PLoS One. 2024 Nov 12;19(11):e0312568. doi: 10.1371/journal.pone.0312568 (PMC11556685; doi:10.1371/journal.pone.0312568)
Supplement: S1 File — (DOCX) [file pone.0312568.s001.docx]

**Consensus on Maturity-Related Injury Risks and Prevention in Youth Soccer: A Delphi Study.**

**Round 1: Group Report/Synthesis of Evidence**

**1 Summary**

- 1. A multidisciplinary group of thirteen (N = 13) applied sport and exercise science experts from England, Wales and Scotland were recruited to the study, however three (N = 3) panellists opted out from the panel at the start of round one. The group comprised of different Sport and Exercise Science disciplines and some combinations of disciplines (Lead Sport Scientists, N = 3; Head of Academy Science and Medicine, N = 6; Head of Physical Performance, N = 1).

1. **Delphi analysis results: Responses relative to the rest of the panel (Theme 1).**

### 2.1 Question 1: Do you agree that injury prevention is the primary reason for capturing maturity related player data?

- 1. **Table 1:** Open response comments to Q1 statement.

| **Responder ID** | **Q1 Verbatim quote** |
| --- | --- |
| 118598008 | To enable key stakeholders to be educated on when best to appropriately manage training load, to reduce the risk of growth-related injuries. |
| 118642203 | A primary reason for my club is to see how far they are into their development in order to make decisions on retention and releasing of players. |
| 118663866 | Injury prevention is part of a complex puzzle around maturity data. Other key reasons include talent identification, talent development. |
| 118706135 | It is only the medical and sport science personnel that use this data. |
| 119024706 | Although injury prevention is an important part, so is individual development. How might we alter the programme to target specific physical qualities during certain stages of growth. Although not using PAH or for retain/release decisions, such information also helps to inform conversations with coaches and recruiters i.e., this player might be struggling to do X physically, but at Y stage in their development this might to be expected, going forwards we would expect to see Z. |
| 119092395 | Dependent on what lens you view it through. Physical staff would say injury prevention, other staff may say performance related / profiling reasons. |
| 119103433 | Data from maturity assessment can be utilised for several purposes, but I don’t think one is a priority over others. For example, it can also be used to guide prescription of athletic development interventions. In regard to injury prevention, I think the data is one factor in determining injury risk, but should be taken in context with any deficits in movement competency as this perhaps considers the mechanism by which PHV increases injury risk. |
| 119131533 | To understand the training load at which the player can complete. |
| 119456003 | It may be linked to performance e.g. poor performance and understanding someone is going through PHV. Also it can be linked to recruitment. |
| 119773168 | It allows us to further enlighten coaches about managing training loads during their PHV. |

### 2.3 Question 2: Do you agree that the Mirwald [2002] method is the most accurate method for assessing age at PHV?

- 1. **Table 2**: Open responses to Q2 statement.

| **Response ID** | **Q2 Verbatim quote** |
| --- | --- |
| 118598008 | I don't believe any methods are of a particularly accurate standard. |
| 118642203 | It is definitely up there. |
| 118663866 | The Mirwald et al. (2002) method can estimate the APHV and does not require longitudinal, this can indicate maturity timing when compared to CA. More specifically, chronological age, stature, body mass and seated stature are used to predict maturity offset using sex-specific equations (Mirwald et al., 2002). The main benefits of this method are it is easy to implement, of low cost and does not require any special equipment (only a stadiometer and scales) (Mirwald et al., 2002). It is important to note, peak leg length growth occurs before or during PHV in the majority of adolescents, 75.6% and 77.6% for girls and boys respectively (Mirwald et al., 2002). The opposite is true for peak trunk height growth which occurs after or during PHV for 71.3% of girls and 83.5% of boys (Mirwald et al., 2002). The Mirwald et al. (2002) equation uses the ratio between leg length and trunk height, which increases pre-PHV, is greatest during PHV and decreases post-PHV. Resultantly, a key limitation of this method is that not all girls and boys grow in that manner, which is reflected by the percentages. Furthermore, this prediction equation has some other inherent limitations. More specifically, the equations have been shown to have systematic errors associated with both chronological age and maturity timing. Comparing predicted versus observed APHV in samples of Polish youth, Malina and colleagues found the maturity offset equation to underpredict APHV in younger children and overpredict APHV in older youth. Further, the prediction errors were substantially magnified in youth who were identified as either early or late maturing (Malina et al., 2016; Malina & Koziel, 2014). The prediction equation, therefore, is only of appropriate accuracy in the narrow age range of 13.00 to 14.99 years and for boys who mature on-time (Malina et al., 2015). As noted, a major limitation of this method is that it is generally inaccurate for early and late-maturing boys and girls. For early-maturing boys, the predicted APHV were later than the measured APHV, whereas, for late-maturing adolescents predicted APHV were earlier than the actual APHV (Kozieł & Malina, 2018). Overall, this equation is only appropriate for boys who are on-time maturity status between 13 and 15 years old. This causes a reduced range of variation in predicted APHV resulting in a central tendency so most adolescents are classified as on-time. Furthermore, in a sample of youth footballers, the maturity offset method did not improve the estimation of PHV compared to the generic age method; however, the percentage of predicted adult height showed better identification of PHV (Parr et al., 2020). |
| 118706135 | My knowledge on more recent research is not as vast therefore unqualified to answer yes or no. |
| 119024706 | Although once popular practice in football, I believe that there is now some concern using this method due to a regression towards the mean. |
| 119092395 | Mirwald or Shearer, difficult to make comparisons between two. |
| 119103433 | I typically use this measurement to predict age-at-PHV. It tends to correspond quite well with other measurements (e.g. if the Mirwald equations identifies someone as circa-PHV, this player will often have a high growth rate) and what I see anecdotally with a players’ stature (e.g. long legs / short trunk of those identified as circa-PHV), but there is often a fluctuation in the value the equation gives you between measurements. |
| 119131533 | Recording standing height, seated height and leg length provides enough data to calculate the PHV. Its reliable and applicable to time management. |
| 119456003 | Only one I’ve used. |
| 119773168 | We use this or the Khamis Roche as it is detailed and takes numerous factors into consideration. |

### Question 3: Do you agree that the Fransen [2018] method is the most accurate method for assessing age at PHV?

- 1. **Table 3**: Open responses to Q3 statement.

| **Response ID** | **Q3 Verbatim quote** |
| --- | --- |
| 118598008 | As above. |
| 118642203 | A method we utilise. |
| 118663866 | Fransen, Bush, et al. (2018) introduced a new model aiming to maintain prediction accuracy and improve prediction in early and late maturers. A limitation of this method was identified by Nevill and Burton (2018) who suggested that there is a mathematical error concerning spuriously high R^2^ values. In their rebuttal, Fransen, Baxter-Jones, and Woodcock (2018) that there is a difference between an explanatory model for hypothesis testing and a predictive model, in the predictive model multicollinearity will not affect the ability of the model to predict. Having said this, this method has yet to be tested to the same extent as the other maturity offset equations. |
| 118706135 | As with all science there is more recent research that puts forward different methods that may be more reliable example (Sanders et al 2021). |
| 119024706 | To be honest I am unfamiliar with this method. |
| 119092395 | Prefer Mirwald / Shearer. |
| 119103433 | I have never used this equation in practice so couldn’t comment on its reliability. |
| 119131533 | From experience the reliability is low |
| 119456003 | N/A |
| 119773168 | N/A |

### 2.7 Question 4: Do you agree that the Moore [2015] methods are the most accurate methods for assessing age at PHV?

2.8 **Table 4**: Open responses to Q4 statement.

| **Response ID** | **Q4 Verbatim quote** |
| --- | --- |
| 118598008 | As above. |
| 118642203 | Multiple papers quote this. |
| 118663866 | As above, see Koziel and Malina 2017. |
| 118706135 | A popular method but does not mean it is the most accurate as with all science new research can be more beneficial but equally can be just fashionable. |
| 119024706 | To be honest I am unfamiliar with this method. |
| 119092395 | Prefer Mirwald / Shearer. |
| 119103433 | I have never used this equation in practice so couldn’t comment on its reliability. |
| 119131533 | Unknown. |
| 119456003 | N/A |
| 119773168 | N/A |

### 2.9 Question 5: Do you agree that the Khamis-Roche [1994] method is the most accurate method for predicting adult height?

2.10 **Table 5:** Open responses to Q5 statement.

| **Response ID** | **Q5 Verbatim quote** |
| --- | --- |
| 118598008 | Most commonly used method to my knowledge, and very well informed and cited method to assess %PAH. |
| 118642203 | It is an old method. |
| 118663866 | Limitations of the Khamis and Roche (1994) method are mainly associated with the measurement of mid-parent height. The biological parent's heights are a key requirement of this equation. This is a limitation as these values may not be available for all adolescent athletes for several reasons. Heights of the biological parents of each player are often self-reported as part of this method which increases error in the estimation as self-reported values also need to be adjusted for over-estimation (Epstein, Valoski, Kalarchian, & McCurley, 1995). A key limitation, which effect all research within this field is the effects of secular trends (Mills et al., 2017). There have been conflicting findings regarding changes to the onset of pubertal events, however, research evaluating data from the current century to those in the 1990s reported the onset of pubertal events was significantly earlier in the current century (Sorensen, Aksglaede, Petersen, & Juul, 2010). Resultantly, research establishing normative values is required, particularly prospective longitudinal studies monitoring PHV in current 21st-century adolescents. |
| 118706135 | Around for 30 years and whilst a tried and trusted method, there are perhaps more accurate methods but there is a cost benefit to some. |
| 119024706 | From the information I have read, and the experts I have heard speak I believe the error range to be too great to confidently predict adult height. Rather than trying to predict adult height, we prefer to use the information to support individual development. |
| 119092395 | Yes, however important to take into account the accuracy of reported parents heights and the bandwidth of which the heights can be. |
| 119103433 | This is certainly the most common measurement used, and the one most discussed in the literature. There is a measurement error with the equation and relies heavily on parents providing accurate heights , unless we measure it ourselves (which isn’t always logistically possible). My cynicism with this method is not so much with the accuracy but how it might be interpreted by coaches. For example if a CB is predicted to be relatively short in stature, are the coaching team more likely to release them or challenge them in different positions?. If it’s the former, I wouldn’t want to put my name to that equation when so many factors can affect the result. |
| 119131533 | Has the power to predict through a long term way. |
| 119456003 | N/A |
| 119773168 | Takes numerous factors into consideration and it seems to work well for PAH. |

### 2.11 Question 6: Do you agree that the Bayley-Pinneau [1952] method is the most accurate method for predicting adult height?

2.12 **Table 6**: Open responses to Q6 statement.

| **Response ID** | **Q6 Verbatim quote** |
| --- | --- |
| 118598008 | This is a method I am unfamiliar with. |
| 118642203 | Things have changed since then. |
| 118663866 | N/A |
| 118706135 | See the previous answer. |
| 119024706 | To be honest I am unfamiliar with this method. |
| 119092395 | Prefer Khamis-Roche. |
| 119103433 | My understanding is that this method overestimates adult height. |
| 119131533 | Unknown. |
| 119456003 | N/A |
| 119773168 | N/A |

### 2.13 Question 7: Do you agree that the collection of player maturity related data can facilitate with injury prevention?

2.14 **Table 7:** Open responses to Q7 statement.

| **Response ID** | **Q7 Verbatim quote** |
| --- | --- |
| 118598008 | Inform coaches of when a player's body will most rapidly change, and increase the likelihood of muscular and bone related injuries. |
| 118642203 | What is needed is what to do during the 85-95% period in order to reduce injury |
| 118663866 | There are some research studies which have demonstrated positive changes after collecting maturity data. |
| 118706135 | Like all data collected it needs to be used for the relevant purpose. |
| 119024706 | Although sports injuries are extremely complex, awareness of PHV and PWV can generate greater individualisation i.e., training/match load modification, change in gym-based exercise selection, increased nutrition support etc. All of which could contribute positively to reduced injury risk. |
| 119092395 | Identifying rapid periods of growth and PHV status can help reduce injury risk and individualize programmes. |
| 119103433 | We know that players circa-PHV and/or with a high growth rate have an increased risk of injury, so collecting that data is certainly useful in identifying those at risk. But I think this should be taken in conjunction with other variables such as overall training load, movement competency / signs of adolescent awkwardness and history of apophysitis, to determine those who may need intervention. |
| 119131533 | To understand where the player is at in relation to PHV, when to adjust training schedules. |
| 119456003 | Mobility targeted to growth related areas during PHV. |
| 119773168 | It allows you to manage training loads during specific times of their growth as well as give them info about muscle imbalances for the gym. |

2.15 **Question 8:** Do you agree that the regular collection (e.g. every 3 months) of maturity related data can facilitate better long-term outcomes (e.g. player progression/selection or injury risk) for a player?

2.16 **Table 8:** Open responses to Q8 statement.

| **Response ID** | **Q8 Verbatim quote** |
| --- | --- |
| 118598008 | In case any short-term rapid spurts have occurred since the last testing date. If it was every 6 months would mean that if this has occurred, it may have been missed since the last testing battery. |
| 118642203 | We do this in our environment. |
| 118663866 | There are some research studies that have demonstrated positive changes after collecting maturity data. |
| 118706135 | As long as consistently collected in repeatable method used then can help in longer term. |
| 119024706 | Three months seems to be a sensible timeframe to ensure regular data, but also prevent 'noise'. If we feel that a player is about to approach, or is going through PHV, we might increase testing frequency to every 6 weeks. |
| 119092395 | Giving the MDT every piece of available information (including growth and maturation data) can help inform contracts, programmes and progression. |
| 119103433 | A snapshot of maturity data is only useful at the time of measurement, whereas longitudinal data can provide high quality detail on the mechanism by which maturation influences physical performance and injury risk, as this can fluctuate over the course of a season. |
| 119131533 | Data suggest no movement of comparison with less than 3 months. The recorded data provides a good understanding of the players progression. |
| 119456003 | Exactly as described within the question. |
| 119773168 | This gives enough time for progression and changes in players. |

### 2.17 Question 9: Do you agree that currently available methods (e.g. predictive equations and medical scanning) for assessing a players maturational status and the timing of specific maturational events are sub-optimal and require improvement?

2.18 **Table 9:** Open responses to Q9 statement.

| **Response ID** | **Q9 Verbatim quote** |
| --- | --- |
| 118598008 | No, however they are typically invasive and so cannot be performed. |
| 118642203 | It is important that there is a simple method for clubs to gather the most accurate data in order to accurately predict PHV. |
| 118663866 | The Khamis Roche method needs to be tested in greater detail. |
| 118706135 | They are sporadic and with such high staff turnover the reliability of data collectable is questionable. |
| 119024706 | I think great improvements have been made in this area over the past 10 years or so, especially with regards to awareness. The current methods provide an estimate and perhaps this is all that is required. Personally, I am a believer in giving every child every chance, with those who 'make it' being due to a myriad of factors. I would be wary of making single factors too predictive in case this discounts an individual who might find a way to cope and excel. I do agree that greater clarity on the best predictive models to use would be useful, alongside how/when to use them. This would drive consistency between clubs (player transfers/bio-banding events etc.) and enhance the application of research. |
| 119092395 | Methods could potentially be viewed as outdated. |
| 119103433 | Predictive equations are not 100% reliable. As players’ futures are often determined with these equations - or certainly form part of retain/release decisions - then we have an ethical responsibility to make these methods as robust and reliable as possible. |
| 119131533 | I believe other methods around parents height/detailed loading must be assessed closer. |
| 119456003 | I think for no cost, current methods are enough. |
| 119773168 | Could be more specific in regards to if they are at the start or end of their PHV. Also further accuracy on PAH. |

1. **Delphi analysis results: Responses relative to the rest of the panel (Theme 2).**

### 3.1 Question 10: Please rank the level of importance of the following maturity-related injury risk factors from an injury prevention perspective in academy soccer players

### 3.2 Accelerated growth rates (e.g., Peak Height Velocity)


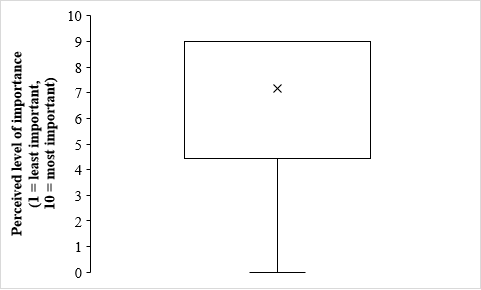


Median = 9

- 1. Upper and lower extremity growth rates (e.g. Arms, Legs, Hands, Feet)


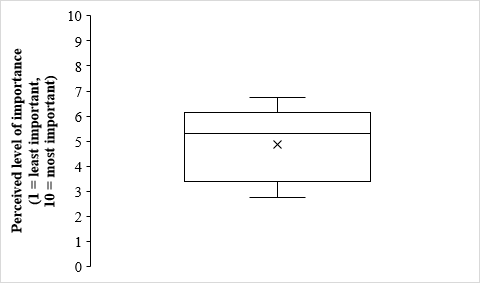


Median = 6

- 1. Muscular strength and flexibility imbalances


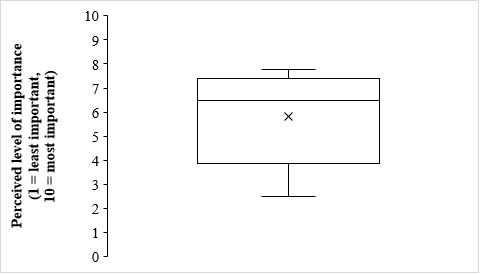


Median = 7

- 1. Abnormal movement mechanics (i.e. biomechanics) or functional movement scores


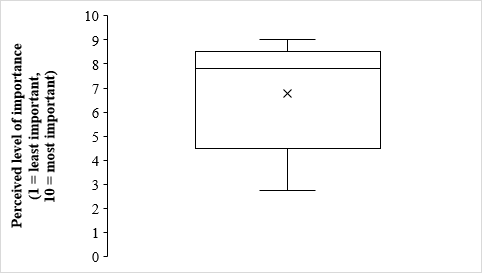


Median = 8

3.6 The period leading up to Peak Height Velocity (i.e. 12 months prior to age at Peak Height Velocity)


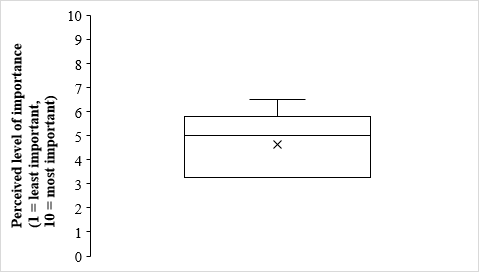


Median = 5

- 1. The period during Peak Height Velocity (i.e. age at Peak Height Velocity)


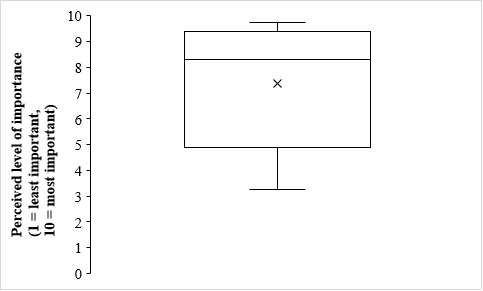


Median = 9

- 1. The period after Peak Height Velocity (i.e. 12 months post age at Peak Height Velocity)


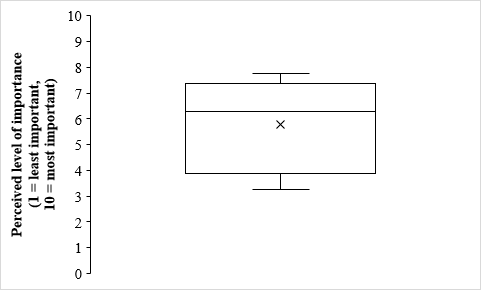


Median = 7

- 1. Fluctuations in lean body mass (i.e. Peak Weight Velocity)


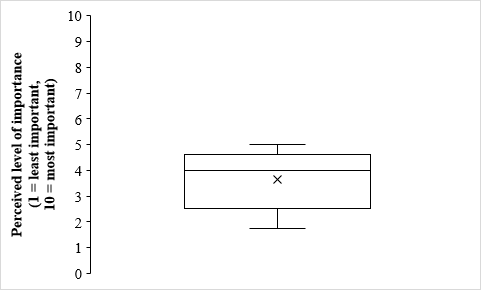


Median = 4

3.10 Previous injury history


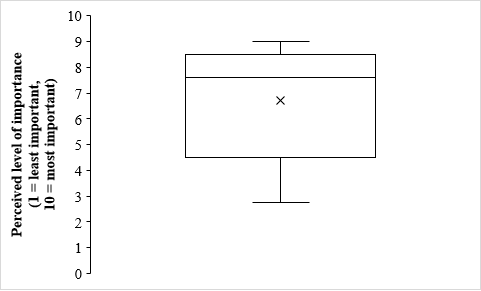


Median = 8


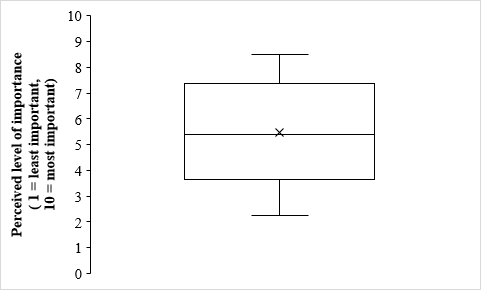
3.11 Individual player maturational timing (i.e. early, on-time, late maturing players)

Median = 5

3.12 A player’s current percentage of predicted adult height (i.e. 80%, 85%, 90%,95% of predicted adult height)


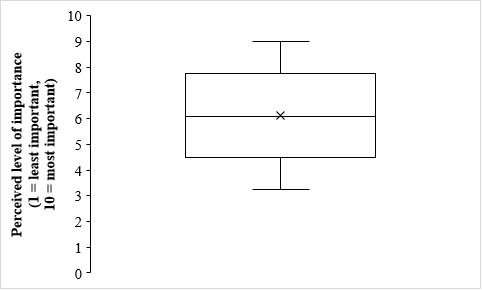


Median = 7

3.13 **Question 10a:** Please include any additional comments such as any statements not included which you think are important.

3.14 **Table 10:** Open responses to Q10a statement.

| **Response ID** | **Q10a Verbatim quote** |
| --- | --- |
| 118598008 | N/A |
| 118642203 | N/A |
| 118663866 | Some of the statements overlap, for example percentage of predicted adult height and PHV. Predicted adult height is a method of measuring the timing of PHV. |
| 118706135 | As long as data collected is consistent over a long period to allow for any trend and patterns to aid in identification. |
| 119024706 | N/A |
| 119092395 | None |
| 119103433 | High training load - accounting for club, school, international, district competition, other sports and PE exposure. |
| 119131533 | The players external activities. |
| 119456003 | N/A |
| 119773168 | N/A |

1. **Delphi analysis results: Responses relative to the rest of the panel (Theme 3).**

4.1 **Question 11:** What long term (e.g. yearly) outcomes does your club hope to achieve through the regular collection (e.g. every 3 months) of maturity related data?

| **Response ID** | **Q11 Verbatim quote** |
| --- | --- |
| 118598008 | To help identify players at immediate risk of injury, and inform coaches of their training load in and out of training, so that these players can be managed by medical staff appropriately. |
| 118642203 | That we can see progression in players growth and ability. |
| 118663866 | Improved training management, talent identification and talent development. |
| 118706135 | Reduce risk of injury primarily in overuse with players during periods of growth. |
| 119024706 | We try to audit squads to inform conversations around player recruitment and retainment. We use the data retrospectively to supplement injury audits. We use longitudinal data as previous case study examples. |
| 119092395 | Monitor changes / significant changes, increase profiling data, implement individual physical/technical and tactical programmes. |
| 119103433 | Identify appropriate athletic development interventions based on biological age, identify late maturers who may need to play down an age group and given them time to develop physically whilst challenging technical skills, identify early maturers who may need to be challenged physically potentially by playing up an age group if their technical ability allows. |
| 119131533 | To understand the players position on the percentage chart, to discuss loading requirements, selection requirements. Comparison of adult height and when will this be achieved. |
| 119456003 | Reduced injury rates and better management of players e.g. More time on pitch if managed correctly. |
| 119773168 | Monitoring their development from the beginning to the end of the year. Managing drastic changes. |

4.2 **Question 11a:** How is maturity related data used to inform injury prevention practices within your club?

| **Response ID** | **Q11a Verbatim quote** |
| --- | --- |
| 118598008 | Gym programmes will be tailored more around those players with a close PHV proximity. Training load is altered only by no-to-little degree. |
| 118642203 | Some players have physio led programs. |
| 118663866 | Identify at-risk individuals. |
| 118706135 | Sudden changes and trends within age groups to inform staff of potential dangers. |
| 119024706 | Information is given to coaches regarding their squads RAE and stage of maturation (i.e., early, late, on-time). Players identified as pre-PHV are monitored more closely. We tend to work more off symptoms but have a lower threshold if we know a player is just before or at PHV. Modifications will be made to training and match loads (volume), with additional supplementary exercises given in the gym. In the future we hope to have an information card for parents with frequently asked questions and homework 'packs' for players (if needed). |
| 119092395 | Decrease in eccentric / plyometric load as a result of rapid growth, reduced technical/tactical load if any growth related symptoms. |
| 119103433 | It is one “flag” in determining injury risk, alongside growth rate, changes in body mass, training load, observations around adolescent awkwardness, reporting of growth-related conditions. Those considered to have an elevated risk will have reduced training load and spend more time with the S&C staff for athletic development interventions. |
| 119131533 | Closer to PHV the loading will be adjusted to minimise growth related risks. |
| 119456003 | Use of a flagging system highlighting players at risk. These are then managed accordingly as per our G&M policy. |
| 119773168 | Pointing out players who are Mid PHV. Informing players will have muscle imbalances and awkwardness with limbs. |

4.3 **Question 11b:** What are the connections between maturity-related changes and increased injury risk in youth academy players?

| **Response ID** | **Q11b Verbatim quote** |
| --- | --- |
| 118598008 | As a player reaches their PHV period, the increase of injury increases due to abnormal muscle and bone development, possible excess loading and stress placed on the body during these times, and players may practise strength/power development when the body is not yet ready for such a demand to be placed on it. |
| 118642203 | Very high as well as playing surface. |
| 118663866 | Rapid change in body tissues. |
| 118706135 | Mainly overuse as continue to demand from players on joints. |
| 119024706 | In my experience a heightened injury risk occurs around PHV due to hormonal changes and alterations in general locomotive skills. It is also logical to suggest that there are greater imbalances between the muscular and skeletal systems through periods of accelerated growth that can alter force production and absorption capabilities. |
| 119092395 | Increased injury risk or risk of growth related symptoms resulting in time-loss. Reduction in performance markers both subjectively and objectively. Potential reduction in technical and tactical performance due to adolescent awkwardness. |
| 119103433 | A player who is circa-PHV is likely to have a high growth rate, resulting in “long levers” and a lag in spatial awareness, resulting in suboptimal movement competency, and therefore poorer landing mechanics or overload of structures, resulting in an increased injury risk. Those with a high growth rate are likely to be rapidly developing bone at epiphyses, causing tension at the tendon attachment, resulting in apophysitis. At the hip, the epiphysis will be the “weak link” so avulsion is more likely than an MTJ tear. A player who is undergoing peak weight velocity will be required to control a greater mass, which may result in overuse injury, particularly bone stress injury as there is likely a lag between body mass and bone mineral density during this period. |
| 119131533 | The greater the PHV the higher the risk of injury. This has been shown through hips, ankles etc reporting to be aware. |
| 119456003 | Tight muscles, pulling on growth sites. Reduced mobility. |
| 119773168 | When players are mid-PHV they are at an increased risk. |

- 1. **Question 11c:** How effective do you believe the current maturity assessment methods employed at your club are in preventing injuries? Please explain your answer

| **Response ID** | **Q11c Verbatim quote** |
| --- | --- |
| 118598008 | Yes, they are successful to a degree. With regular 3-month G+M testing periods, FMS interventions, and individual management of gym loads, there have been a small number of growth-related injuries within the academy. |
| 118642203 | It is difficult because the coaches have minimal buy-in. |
| 118663866 | Moderate. |
| 118706135 | Not very good as a constant revolving door of personnel therefore data is being overlooked or late being reported after events. |
| 119024706 | We are currently undertaking a project in this area. We need to ensure that all data collected is as accurate as possible (i.e., taken by a qualified/experienced practitioner using standards and calibrated equipment. Data storage and visualisation also needs to be appropriate including the use of correct formulas). We need to get better at using technology to flag key milestones in players' growth/development and have clearer interventions when we know changes are taking place. The overall programme has been reviewed to try and address global/generic issues, but we now need to move towards more individualised/specific processes. |
| 119092395 | Not effective. This is due to lack of connection with technical staff to reduce pitch based load. |
| 119103433 | It is a relatively new relationship, and it is often hard to effect change pre-emptively, so it is often used in hindsight to explain to coaches why an injury has happened. We are hopeful that as the relationship develops and we justify the use of maturation data, we will be able to intervene earlier. |
| 119131533 | I believe the process is handled professionally on recording, tracking and have the control and support of the academy manager on when to adjust a programme. |
| 119456003 | Moderately effective. |
| 119773168 | I believe they are effective especially when coaches are informed of it. |

4.5 **Question 11d:** Are you satisfied with the current data collection practices of maturity related data at your club? Please explain your answer

| **Response ID** | **Q11d Verbatim quote** |
| --- | --- |
| 118598008 | Yes as I feel I have took some time to find the way in which I would like to collect and feedback data. However now I have become accustomed to my way of working and have become more able with the collection of data. |
| 118642203 | No, Would be more beneficial to know what injury prevention strategies to employ. |
| 118663866 | Yes. |
| 118706135 | No- see previous answer. |
| 119024706 | Please see above. |
| 119092395 | Yes, however work to be done with regards to consistency and how information informs practice. |
| 119103433 | We are split over several sites so it is difficult to obtain the measurement regularly enough to gain appropriate insight. |
| 119131533 | Yes, every 3 months it is recorded to ensure all databases are up to date and coaches are informed. |
| 119456003 | Yes I think they are sufficient. |
| 119773168 | Yes I believe them to be thorough and give good insight for all players. |

4.6 **Question 11e:** How would you change the maturity related injury data collection practices at your club? And if not, what would you do differently?

| **Response ID** | **Q11e Verbatim quote** |
| --- | --- |
| 118598008 | I would have all players complete both mother and father parental heights upon signing for the club, so that this information can be easily processed when calculating %PAH. |
| 118642203 | More bio-banded sessions and games. |
| 118663866 | Further testing of the KR method, collection of ultrasound methods. |
| 118706135 | Stick to one method of collecting and reporting and have incorporated within the academy working practice as a component that is used as part of. |
| 119024706 | We are currently in the process of moving to Kitman Labs. This should improve security, transparency and visualisation. |
| 119092395 | Instead of email communication, report verbally and visually with MDT and figure out individual plans as a result. |
| 119103433 | Greater staff provision to ensure we collect data often enough to aid in training prescription at an individual level. |
| 119131533 | Provide a meeting with all staff to highlight where each player is at. Have a club wide understanding. |
| 119456003 | N/A |
| 119773168 | N/A |

4.7 **Question 11f:** Do you have any feedback of any existing methods that are used for maturity related data collection in your club? Please explain your answer

| **Response ID** | **Q11f Verbatim quote** |
| --- | --- |
| 118598008 | %PAH has been a positive method for me, and one I use when feedbacking growth-related data to coaches and staff. I feel that Maturity Offset methods, whilst valuable information, I am unsure as to which has been deemed the most accurate, and so tend to avoid feedbacking these when creating individual reports for players. |
| 118642203 | We use standing and seated height as well as current weight to predict PHV. |
| 118663866 | N/A |
| 118706135 | That these are not relevant as keep changing, therefore coaches and other stakeholders are not seeing as useful. |
| 119024706 | N/A |
| 119092395 | Simple data collection, Khamis-Roche and Mirwald method + data visualisation. |
| 119103433 | We need to make data visualisations easy to interpret for coaches and senior management, which could help in understanding the information and engaging them with maturity data. |
| 119131533 | The process can be rushed due to number of players/staff ratio. To ensure a smooth transition more staff is needed to record sufficiently |
| 119456003 | N/A- No need to change currently. |
| 119773168 | N/A |

4.8 **Question 11g:** Does your club utilise any invasive methods (i.e., medical imaging) for maturity-related data collection? If so, please state which invasive methods are used below.

| **Response ID** | **Q11g Verbatim quote** |
| --- | --- |
| 118598008 | No. |
| 118642203 | No. |
| 118663866 | If there is medical rationale. |
| 118706135 | None used. |
| 119024706 | No. |
| 119092395 | No, however club now has a DEXA scan so may be a potential avenue in the future, with parental consent. |
| 119103433 | No. |
| 119131533 | No. |
| 119456003 | No. |
| 119773168 | No. |

4.9 **Question 11h:** In your opinion, are invasive methods (i.e., medical imaging) more useful and informative than non-invasive methods (i.e. predictive equations) for maturity related data collection? Please explain your answer

| **Response ID** | **Q11h Verbatim quote** |
| --- | --- |
| 118598008 | Yes, as they give a clear and more appropriate assessment of where a player currently is with their maturation. With non-invasive methods, there is much deliberation about the predictive equations math, the most accurate methods, and 'adjusting for over/under estimation' with regards to the %PAH. However, with invasive methods, you have a more 'black and white' picture of where the player is, based on objective data results. |
| 118642203 | Potentially but we don't have the facilities for that. |
| 118663866 | It depends, they could provide clearer information in some case, I don't think they are required for all cases. |
| 118706135 | Unsure on how to answer as not enough knowledge about these methods, I guess it comes down to cost benefit. |
| 119024706 | There might be a need in rare cases (i.e., players from overseas with limited information around DOB etc.), but in general I do not think that there is a need for invasive methods. Again, I think estimates are okay because LTAD is multifactorial with singular components only contributing to the overall picture. |
| 119092395 | Both useful however invasive methods potentially more accurate, also potentially less practical. |
| 119103433 | They may be more reliable than non-invasive methods but the ethical implications and financial & logistical considerations mean the harms probably outweighs any benefits. |
| 119131533 | In my opinion medical imaging should only be used if a player is going highly graded pain / discomfort due to the percentages being off / development accuracy. |
| 119456003 | I have never used these so can’t comment. |
| 119773168 | Due to volume of players and limited time non invasive measures are better. |

4.10 **Question 11i:** In your opinion, is ‘bio-banding’ (i.e. categorising players into specific maturity-related groupings) an effective method of injury prevention for maturity related injuries? Please explain your answer.

| **Response ID** | **Q11i Verbatim quote** |
| --- | --- |
| 118598008 | I think it can be in a physical development environment. If you have 'Pre-PHV, Circa-PHV, and Post-PHV' maturing groups of players, the training load and tasks can be tailored accordingly, based on what group you are working with. This may mean sessions have more speed endurance, or strength-based components, which may not be suitable for other-PHV groups. I think it gives a sense that training can be more individualised and appropriate to each players current state of maturation. |
| 118642203 | Yes as it helps develop players at all ages and stages. |
| 118663866 | Yes. |
| 118706135 | Not a simple yes or no answer, I feel it has its uses at very young age but as they get older, it is something that experience in life and the game and therefore has to be introduced. |
| 119024706 | I don't think bio-banding is useful for injury prevention because I would only use it for part of the programme i.e., for a short period or certain tournaments/festivals. I believe bio-banding to have many benefits (including psychosocial) but injury prevention is not one. |
| 119092395 | No. I believe bio-banding is more of a method of increasing technical / tactical performance relative to their biological age. |
| 119103433 | Yes. It may protect late maturers from the physical nature of early maturers, and the external load they are exposed to, which could reduce injury - although I think it is primarily used as a method for technical development. |
| 119131533 | Yes, you're able to compare technical ability to the players. Through understanding the technical ability, post PHV the coaches / recruitment will already understand that technical skill level is a high for a certain player. |
| 119456003 | I haven’t used this for the sole purpose of injury prevention previously. |
| 119773168 | Yes they are playing players their size so less likely to get injured from bigger players. |
