# Supplementary material for: Consensus on maturity-related injury risks and prevention in youth soccer: A Delphi study
Source: PLoS One. 2024 Nov 12;19(11):e0312568. doi: 10.1371/journal.pone.0312568 (PMC11556685; doi:10.1371/journal.pone.0312568)
Supplement: S2 File — (DOCX) [file pone.0312568.s002.docx]

**Consensus on Maturity-Related Injury Risks and Prevention in Youth Soccer: A Delphi Study.**

**Round Two: Background Report**

**1 Background to round two:**

Many thanks to all our panellists for your ongoing co-operation and participation in the study. It has been a fascinating process looking at the pattern of data and reading your comments. We are excited about the potential this project has for moving the field forward. Consideration of responses from round one led to consensus on some items, as well as ideas for dropping/rewording/combining items to aid the Delphi process.

In round one, there was a consensus (100%) that the regular collection of maturity-related data can aid with injury prevention and facilitate better long-term outcomes regarding player selection and development. Furthermore, there was also a large agreement (70%) that currently available methods (i.e. predictive equations) for assessing the maturational status and timing of youth players are sub-optimal and require improvement. For maturity-related injury risk factors, there was a perceived higher importance (score ≥ 7) for accelerated growth rates, muscle/strength flexibility imbalances, abnormal movement mechanics, the period during and after (i.e. 12 months) peak height velocity, previous injury history and a player’s maturity status as a percentage of predicted adult height. The least important maturity-related injury risk factors (score ≤ 5) were group maturity status, fluctuations in lean body mass and the period before (i.e. 12 months) peak height velocity.

There was also some surprising evidence from round one to suggest that maturity-related data collection isn’t captured solely for injury prevention, but instead may have ‘other’ holistic benefits. This contrasts previous work which has suggested that the primary reason for maturity-related data collection is to facilitate with injury prevention practices and outcomes for academy players. There was also an apparent gap in practitioner knowledge surrounding the available methods for assessing age at peak height velocity and percentage of predicted adult height. There was a disparity between the level of agreement and verbatim quotes surrounding the accuracy of maturity-related data collection methods. Lastly, there seemed to be a lack of agreement surrounding the primary use of ‘bio-banding’, with some references to injury prevention as a way of protecting late maturing players and other references suggesting it is as a talent development method, by challenging players particularly those who are early maturing.

In this document, we list a revised set of **eighteen statements**, based on the responses from round one, which you will be asked to rate in round two (**via an online questionnaire to be sent separately)**. Your ratings of these items will indicate whether there is panellist consensus from a validation perspective (i.e. the extent to which you agree with the statement). Also, we will be asking all panellists to provide some additional explanation and rationale surrounding their level of agreement with our proposed statements. You will also be provided with the opportunity to propose alternative wording or challenge the statements if you feel we have misrepresented research knowledge **(use the comments box on the online questionnaire)**.

Ultimately, this additional information will be incorporated into the final report for items where there is reasonable consensus (≥ 70%). Please be assured that all respondents will remain anonymous and individual comments can only be identified by the main author (Joe Sullivan).

**2 New items for round two:**

We had planned to simply retain items with good agreement, but we have gone beyond this on the basis of comments made by panellists and have dropped, collapsed and reworded many items. The whole process should be transparent – the group report (sent in a previous email) gives the distribution of ratings and the comments, and this document explains the relationship between items in round one and round two. However, it is complex, so please feel free to contact the main author (Joe Sullivan) if you are unsure about how we have developed these statements presented in round two from the responses in round one. The round two items are grouped into broad categories (maturity data collection purposes, the education/culture of data collection methods, disparity between subjective scores and verbatim quotes for data collection methods, injury prevention solutions, maturity-related injury risk factors and bio-banding). We propose the following eighteen statements in round two:

1. Reasons for the collection of maturity-related data include concerns about overuse/growth related injuries and to identify players at immediate risk of injury.
2. Players with deficits in movement efficiency are at greater risk of growth-related injuries.
3. We have only limited ability to predict which players with deficits in movement efficiency will go on to experience poorer long-term outcomes.
4. Functional assessments that explore “adolescent awkwardness” seem a promising approach. In principle, it may help performance staff understand the mechanisms by which deficits in movement competency around PHV increases injury risk.
5. Maturity-related data allows performance staff to monitor and adjust training load especially for those players closer to PHV.
6. Growth and maturity data is used to inform decisions around player selection/deselection or player recruitment.
7. Growth-related data can be used to identify both early and late maturing players and determine whether players need to play ‘up’ or ‘down’ an age group.
8. Performance staff/sport scientists in academy environments have sufficient education and available support to plan and implement interventions for players with growth-related conditions (e.g., Severs disease, Osgood-Schlatter’s).
9. Performance staff/sport scientists in academy environments have sufficient knowledge and expertise when assessing growth-related conditions and are comfortable using common maturity assessment methods [e.g. Khamis-Roche, 1994; Mirwald, 2002].
10. The Mirwald [2002] equation is only appropriate for use with boys who are on-time in their maturity status between the age of 13-15 years.
11. Current and popular assessments [e.g., Khamis-Roche, 1994] do not show a clear growth and maturity profile due to limitations with the requirement of mid-parent height.
12. Maturity-related data needs to be presented in a manner that coaches will understand, due to the consequences of data misinterpretation on player development.
13. Medical scanning techniques could provide greater reliability, validity and sensitivity for maturity-related assessment, but non-invasive methods can provide complimentary information.
14. Players who are pre or circa-PHV would benefit from an increased frequency of maturity and injury screening assessments from 12 week to 6-week intervals.
15. Longitudinal maturity-related data collection is preferable as it allows for a more accurate assessment of maturation and its effects on injury risk over the course of the season(s).
16. Accelerated growth rates, imbalances between muscular strength and flexibility, abnormal movement mechanics, the period during and after age at PHV and a players maturity status as a percentage of adult height are the highest priority maturity-related injury risk factors.
17. Training load management and S&C interventions are the most effective strategies to limit the effect of maturity-related injury risk factors.
18. Better understanding of the full application of bio-banding and its wider uses are needed for performance staff.

The link to the second-round questionnaire will be sent out via email in due course. All panellists will be given a maximum of **four weeks** to complete the questionnaire. Please contact the main author Joe Sullivan (J.F.Sullivan@2022.ljmu.ac.uk) or Simon Roberts (S.Roberts2@ljmu.ac.uk) if you have any further questions about the study or if you have issues with your involvement in the study.
