# Supplementary material for: Consensus on maturity-related injury risks and prevention in youth soccer: A Delphi study
Source: PLoS One. 2024 Nov 12;19(11):e0312568. doi: 10.1371/journal.pone.0312568 (PMC11556685; doi:10.1371/journal.pone.0312568)
Supplement: S3 File — (DOCX) [file pone.0312568.s003.docx]

**Consensus on Maturity-Related Injury Risks and Prevention in Youth Soccer: A Delphi Study.**

**Round 2: Group Report/Synthesis of Evidence**

**1 Summary**

- 1. A multidisciplinary group of ten (N = 10) applied sport and exercise science experts from England, Scotland and Wales participated in round two. The group comprised different disciplines and some combinations of disciplines (Lead Sport Scientist, N = 3; Head of Academy Science and Medicine, N = 6; Head of Physical Performance, N= 1).

Eighteen (N = 18) statements were generated based on the results and comments by all panellists in round one. All panellists were asked to state their level of agreement with each statement on a Likert-scale (1 = strongly disagree, 10 = strongly agree). Furthermore, for panellists that agreed with the proposed statement but not on its wording were invited to suggest alternatives via an open-text comment box for each statement.

From the eighteen (N = 18) statements, consensus (≥70%, median score = 7/10) was achieved on thirteen (N = 13) statements. The mean, median and interquartile range for these statement scores are presented below.

1. **Results**
   1. **Statement 1:** Reasons for the collection of maturity-related data include concerns about overuse/growth related injuries and to identify players at immediate risk of injury.


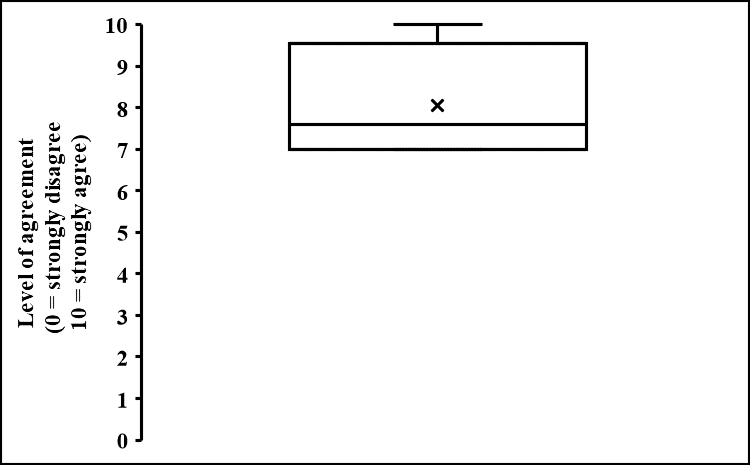


**Mean = 8**

**Median = 7**

**IQR = 3**

- 1. **Statement 2:** Players with deficits in movement efficiency are at greater risk of growth-related injuries.


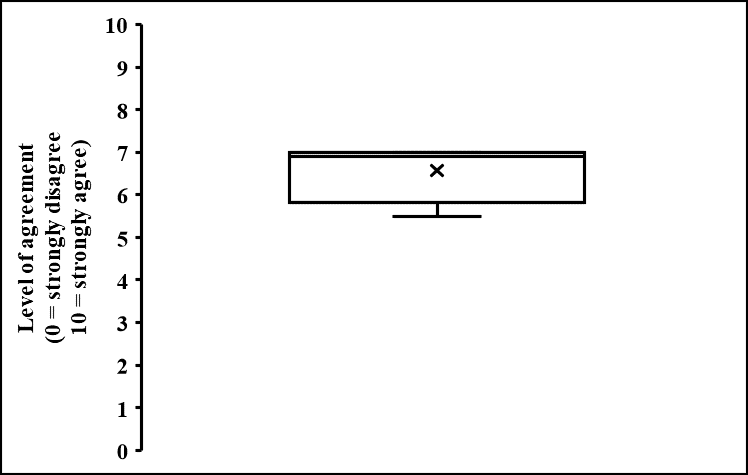


**Mean = 7**

**Median = 7**

**IQR = 2**

- 1. **Statement 3:** We have only limited ability to predict which players with deficits in movement efficiency will go on to experience poorer long-term injury risk outcomes.


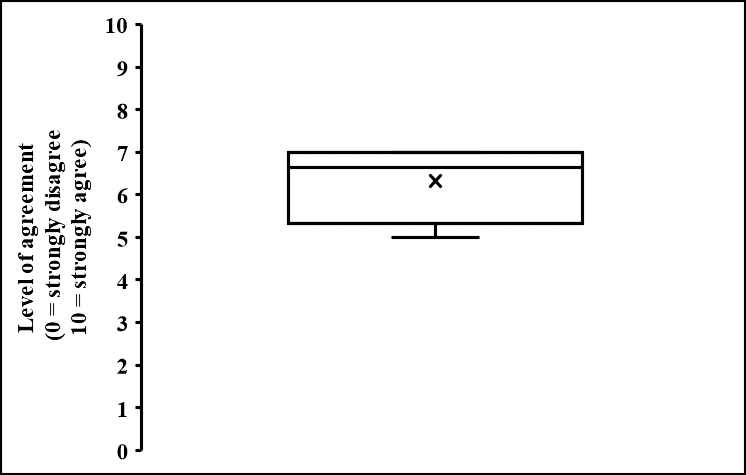


**Mean = 6**

**Median = 7**

**IQR = 2**

- 1. **Statement 4:** Functional assessments that explore “adolescent awkwardness” seem a promising approach. In principle, it may help performance staff understand the mechanisms by which deficits in movement competency around PHV increases injury risk.


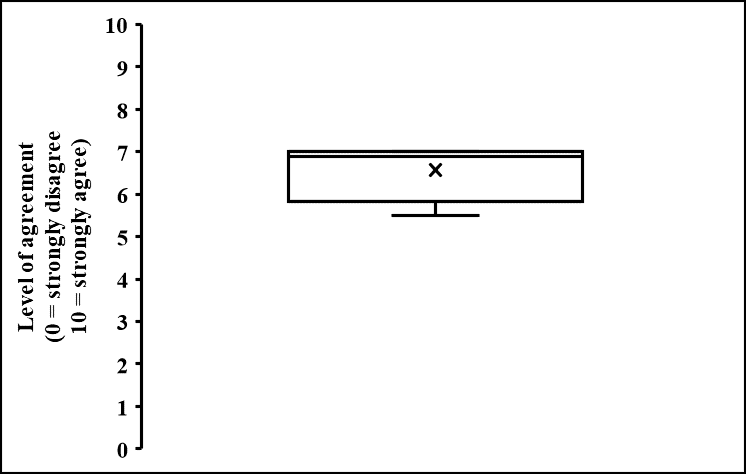


**Mean = 7**

**Median = 7**

**IQR = 2**

- 1. **Statement 5:** Maturity-related data allows performance staff to monitor and adjust training load especially for those players closer to PHV.


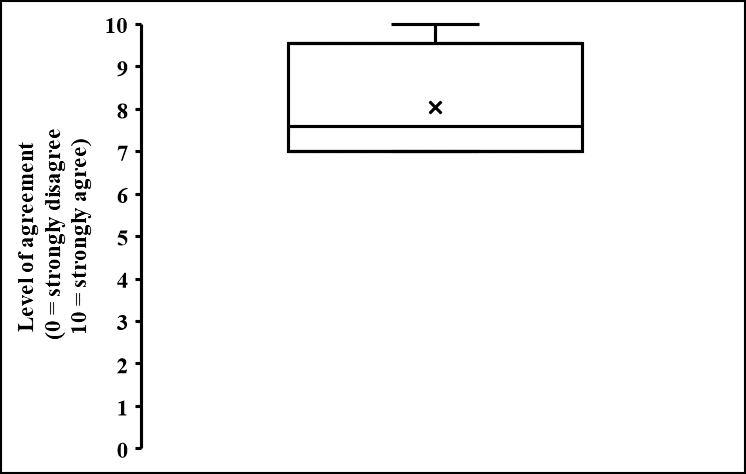


**Mean = 8**

**Median = 7**

**IQR = 3**

- 1. **Statement 6:** Growth-related data can be used to identify both early and late maturing players and determine whether players need to play ‘up’ or ‘down’ an age group.


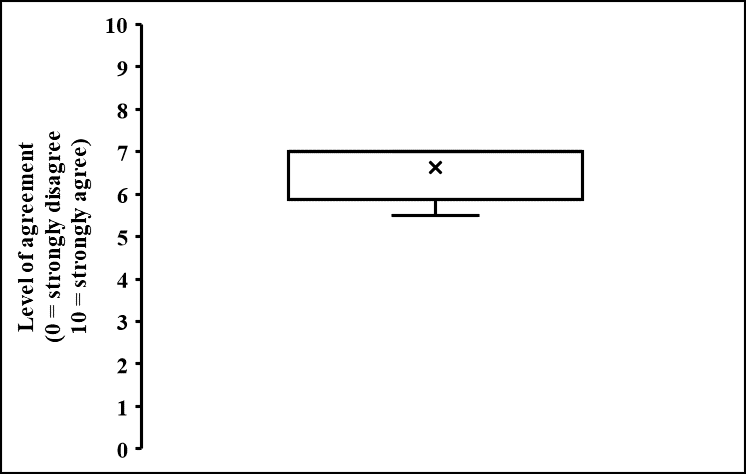


**Mean = 7**

**Median = 7**

**IQR = 2**

- 1. **Statement 7:** Maturity-related data needs to be presented in a manner that coaches will understand, due to the consequences of data misinterpretation on player development.


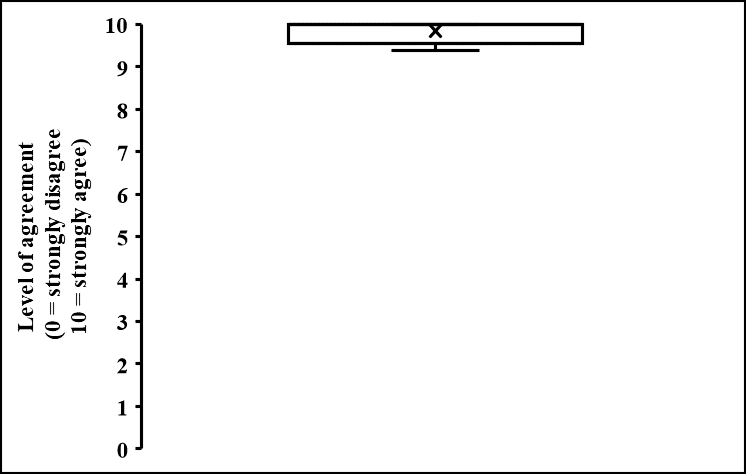


**Mean = 9**

**Median = 10**

**IQR = 0**

- 1. **Statement 8:** Medical scanning techniques could provide greater reliability, validity and sensitivity for maturity-related assessment, but non-invasive methods can provide complimentary information.


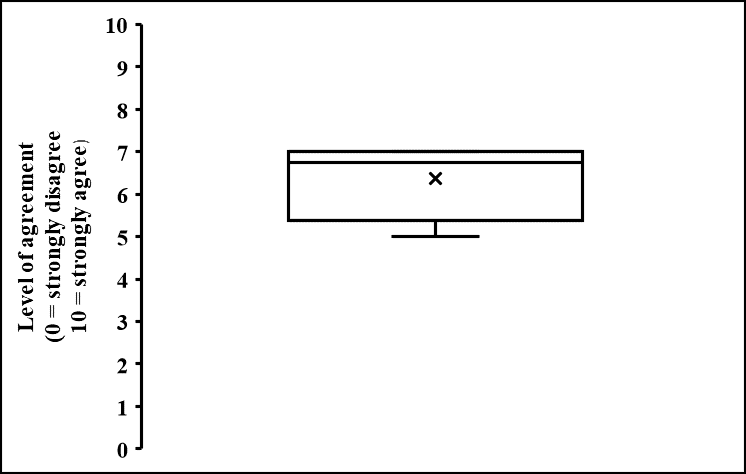


**Mean = 7**

**Median = 7**

**IQR = 2**

- 1. **Statement 9:** Players who are before or during PHV would benefit from an increased frequency of maturity and injury screening assessments from 12-week to 6-week intervals.


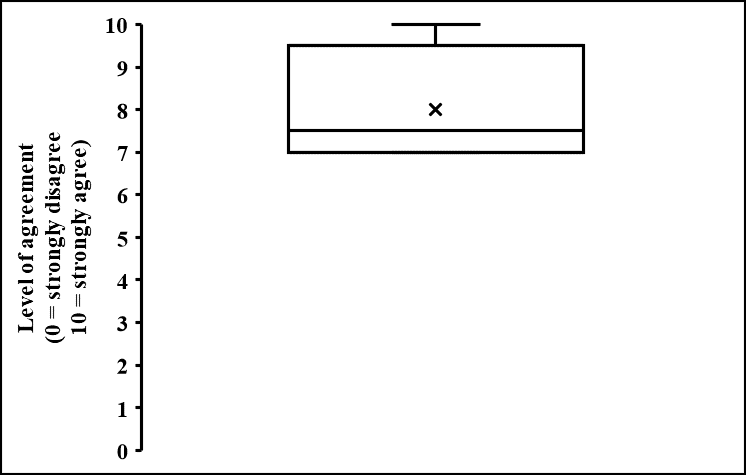


**Mean = 8**

**Median = 7**

**IQR = 3**

- 1. **Statement 10:** Longitudinal maturity-related data collection is preferable as it allows for a more accurate assessment of maturation and its effects on injury risk over the course of the season(s).


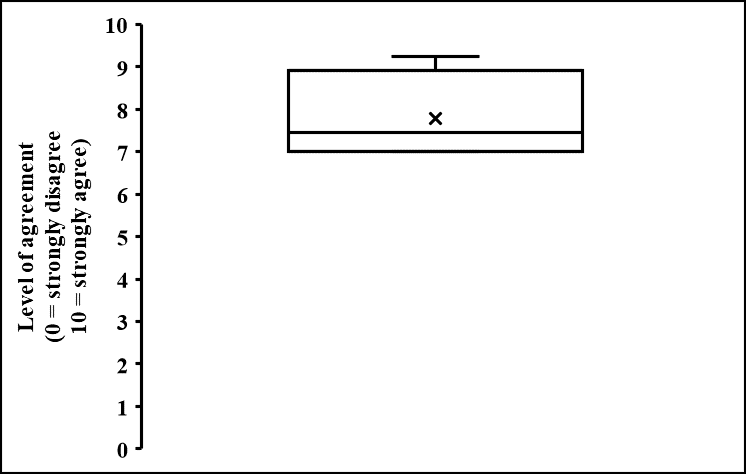


**Mean = 8**

**Median = 7**

**IQR = 2**

- 1. **Statement 11:** Accelerated growth rates, imbalances between muscular strength and flexibility, abnormal movement mechanics, the period during and after age at PHV and a players maturity status as a percentage of adult height are the highest priority maturity-related injury risk factors.


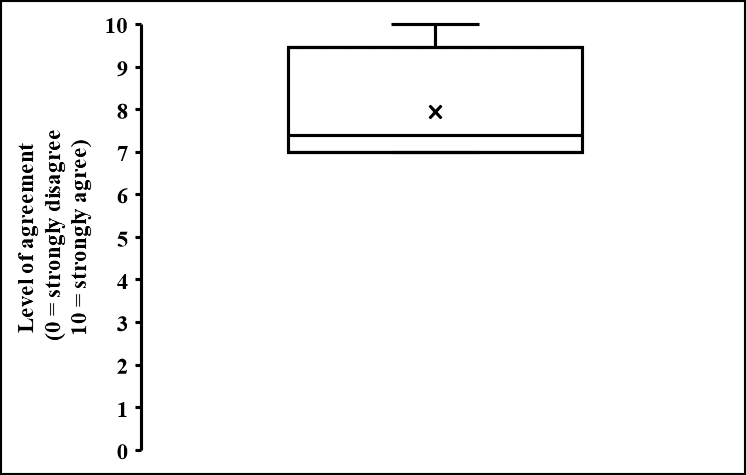


**Mean = 8**

**Median = 7**

**IQR = 3**

- 1. **Statement 12:** Training load management and S&C interventions are the most effective strategies to limit the effect of maturity-related injury risk factors.


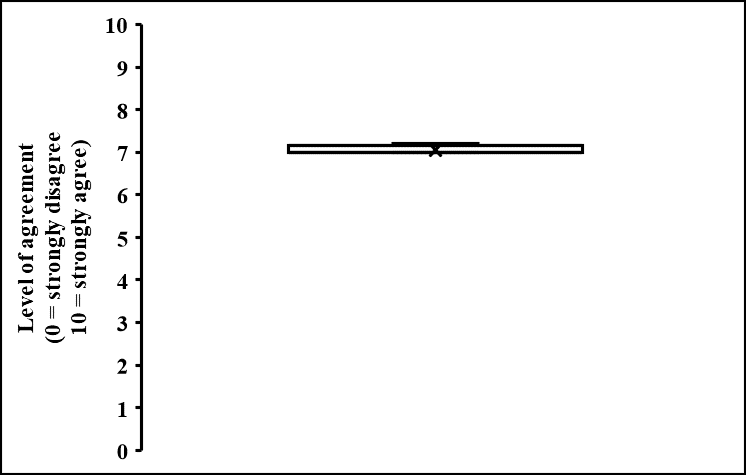


**Mean = 7**

**Median = 7**

**IQR = 0**

- 1. **Statement 13:** Better understanding of the full application of bio-banding and its wider uses are needed for performance staff.


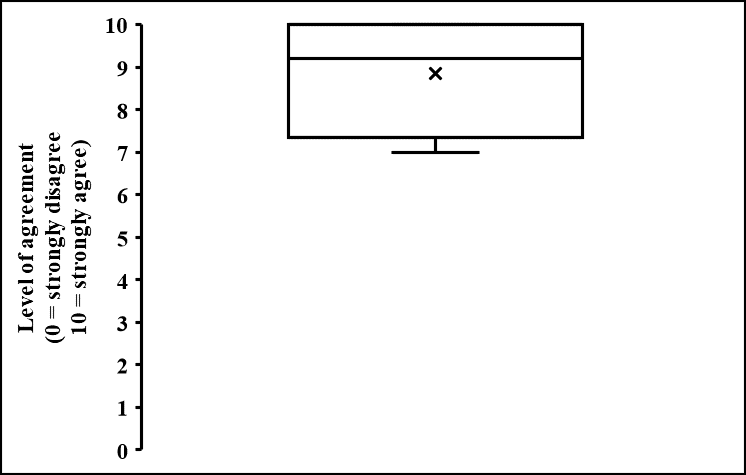


**Mean = 8**

**Median = 10**

**IQR = 3**
