# Supplementary material for: Consensus on maturity-related injury risks and prevention in youth soccer: A Delphi study
Source: PLoS One. 2024 Nov 12;19(11):e0312568. doi: 10.1371/journal.pone.0312568 (PMC11556685; doi:10.1371/journal.pone.0312568)
Supplement: S4 File — (DOCX) [file pone.0312568.s004.docx]

**Consensus on Maturity-Related Injury Risks and Prevention in Youth Soccer: A Delphi Study.**

**Round Three: Background Report**

**1 Background to Round Three:**

Once again, we extend our sincere gratitude to all our panellists for their patience and ongoing participation in the study. Having analysed the scores and comments from the eighteen (N = 18) statements proposed in round two, consensus (≥70%, median score = 7/10) was achieved for thirteen (N = 13) statements. The full analysis can be found in the round two synthesis document.

The research team initially intended to re-send the remaining five (N = 5) statements back to all panellists in round three, in an attempt to achieve consensus (≥70%). However, having re-considered the nature of the remaining statements, it has now been decided by the research team that two (N = 2) of the statements will be removed ahead of round three which are listed below:

*“The Mirwald [2002]) equation is only appropriate for use with boys who are on-time in their maturity status between the age of 13-15 years.”*

*“Current and popular assessments [e.g., Khamis-Roche, 1994] do not show a clear growth and maturity profile due to limitations with the requirement of mid-parent height.”*

The research team thought that these statements placed a requirement on panellists to have an extensive knowledge around the application of these specific methods and their respective limitations. It may also be apparent that some of the professional clubs that our panellists work at, may not use these specific methods for assessing maturity status and timing in their youth players and therefore it would be inappropriate to ask them to score the statement again. There was also a risk of panellists not changing their scores from round two, with a median score of 5-6 (neutral) for both statements, which would lead to further unnecessary rounds in the Delphi process.

**2 New items for Round Three:**

The research team have acknowledged the comments from each panellist from round two. For the statements that achieved consensus from the previous round, the research team will attempt to improve the wording of these statements, having considered the comments made by panellists and these will be presented in round three. However, panellists **will** **not** be asked to rate their level of agreement with these statements. Instead, panellists will be asked if they are satisfied with the wording of the newly proposed statement via a ‘Yes’ or ‘No’ sub-question. Another open-text response box will be provided with each statement to invite panellists to suggest alternative wordings for these statements if they are unsatisfied.

Three statements will be re-sent in round three, in an attempt to achieve consensus. These statements will be re-worded and amended, based on the comments received by panellists during round two for these items. The following statements that will be re-sent are listed below:

“*Growth and maturity data is used to inform decisions around player selection/deselection or player recruitment.”*

“*Performance staff/sport scientists in academy environments have sufficient education and available support to plan and implement interventions for players with growth-related conditions (e.g., Severs disease, Osgood-Schlatter’s).”*

*“Performance staff/sport scientists in academy environments have sufficient knowledge and expertise when assessing growth-related conditions and are comfortable using common maturity assessment methods [e.g. Khamis-Roche, 1994; Mirwald, 2002].”*

The median scores for these statements ranged between 5-6 (neutral). However, following some insightful comments made by panellists during round two, the research team feel that with some amendments, consensus could be achieved for these statements.

For these three statements, panellists will be asked to rank their level of agreement for the newly proposed statement on a Likert-scale, similar to the previous round (1 = strongly disagree, 10 = strongly agree). Panellists who agree with the statement but not on its wording will again be invited to suggest alternatives via an open-text response.

The research team are hopeful that the study will conclude after the completion of round three, having achieved consensus on a large proportion of statements in the previous round. An email will be sent in due course to each panellist containing a unique username, password and URL link to complete survey. A maximum of **four weeks** will be allocated to complete round three of the Delphi poll. For any panellists who wish to withdraw from the study at this stage, please email the lead author Joe Sullivan (J.F.Sullivan@2022.ljmu.ac.uk) or Simon Roberts (S.Roberts2@ljmu.ac.uk).
