# Supplementary material for: Consensus on maturity-related injury risks and prevention in youth soccer: A Delphi study
Source: PLoS One. 2024 Nov 12;19(11):e0312568. doi: 10.1371/journal.pone.0312568 (PMC11556685; doi:10.1371/journal.pone.0312568)
Supplement: S5 File — (DOCX) [file pone.0312568.s005.docx]

**Consensus on Maturity-Related Injury Risks and Prevention in Youth Soccer: A Delphi Study.**

**Round 3: Group Report/Synthesis of Evidence**

**1 Summary**

- 1. A multidisciplinary group of nine (N = 9) sport and exercise science experts from England, Scotland and Wales participated in Round Three. Unfortunately, one (N = 1) panellist who had participated in the previous two rounds did not respond to the final survey.

Thirteen (N = 13) statements gained consensus from round two (≥70%, median score = ≥7/10). However, following some insightful comments made by all panellists, the research team re-worded these statements to improve their validity. These statements were re-sent in round three and panellists were asked to state whether or not they agreed with the wording of these statements via ‘Yes’ or ‘No’ responses. Those who did not agree with the wording of these statements were invited to suggest alternatives via the open-text response box. The level of agreement for these statements is presented below.

1. Reasons for the collection of maturity-related data include concerns about overuse growth related injuries and to identify players at immediate or future risk of injury.
2. Players with deficits in movement efficiency may demonstrate a greater risk of growth-related injuries, however more research is needed given the quality of current evidence.
3. It is implausible to predict which players with deficits in movement efficiency will go on to experience poorer long-term injury risk outcomes. This could be improved with better equipment and education.
4. Functional assessments that explore “adolescent awkwardness” seem a promising but under investigated approach. In principle, it may facilitate conversations with performance staff to help them understand the mechanisms by which deficits in movement competency around PHV increases injury risk and can subsequently influence on-pitch performance.
5. Maturity-related data allows performance staff to monitor and adjust training load especially for those players closer to PHV. However, it should be conducted in a way that considers the individual and their environmental context.
6. Growth-related data can be complemented with performance-related data to identify both early and late maturing players and also to determine whether players need to play ‘up’ or ‘down’ an age group.
7. Maturity-related data needs to be presented in a manner that coaches will understand, due to the consequences of data misinterpretation on player development, selection and training load management.
8. Medical scanning techniques provide greater reliability, validity and sensitivity for maturity-related assessments, but are unlikely to be used in a real world setting due to ethical and financial implications.
9. Players who are before or during PHV, would benefit from an increased frequency of maturity and injury screening assessments from 12-week to 6-week intervals. This could help to closely monitor the physiological processes associated with an increased risk of injury, providing that measurements are taken accurately.

1. Longitudinal maturity-related data collection is preferable as it allows for a more accurate assessment of maturation and its effects on injury risk over the course of the season(s), as well as identifying players who are at an increased risk of injury.
2. Accelerated growth rates, imbalances between muscular strength and flexibility, abnormal movement mechanics, the period during and after age at PHV, reductions in neuromuscular control and a players' maturity status (% predicted adult height) are the highest priority maturity-related injury risk factors.
3. Multidisciplinary approaches towards training/game load management, S&C interventions and consideration of injury history are the most effective strategies to limit the effect of maturity-related injury risk factors.
4. It is unrealistic for practitioners to use bio-banding as a method to reduce injury risk without greater training and research dissemination.

A further four (N = 4) statements were also re-sent during round three, in a final attempt to achieve consensus on these items. Consensus (≥70%, median score = ≥7/10) was achieved for three (N = 3) of these statements, with one (N = 1) statement remaining neutral (median score = 5). The mean, median and interquartile range for these statement scores are presented below.

1. Growth and maturity data can inform decisions around player selection/deselection, recruitment and profiling for positional requirements until the player is aged 16-18 years.


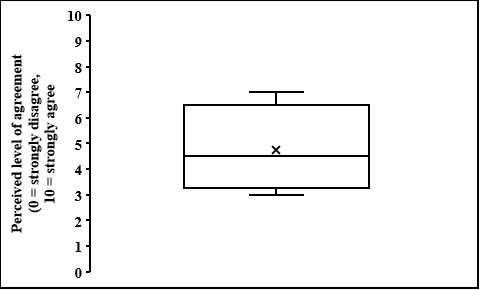


**Mean = 5**

**Median = 5**

**IQR = 4**

1.
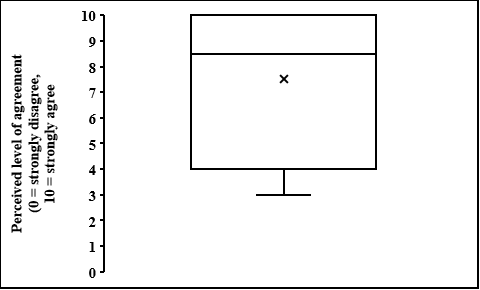
Additional training and education is required surrounding the prescription of interventions for academy players with growth-related conditions such as Severs disease or Osgood-Schlatter’s.

**Mean = 9**

**Median = 10**

**IQR = 7**

1. Performance/sports science staff in academy environments have sufficient knowledge and expertise of taking growth-related measurements and using common maturity assessment methods in practice (e.g. Khamis-Roche, 1994; Mirwald, 2002) to determine a players’ maturity status and the timing of the adolescent growth spurt.


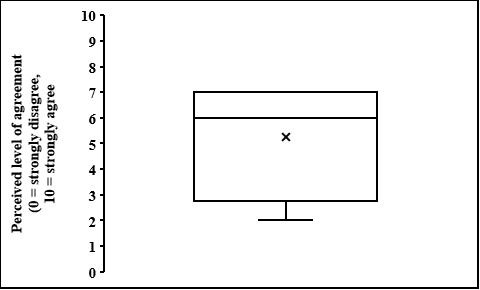


**Mean = 6**

**Median = 7**

**IQR = 2**

1. Apophysitis conditions around the hip are more difficult to diagnose than apophysitis conditions around the foot and ankle and require a specialist assessment.


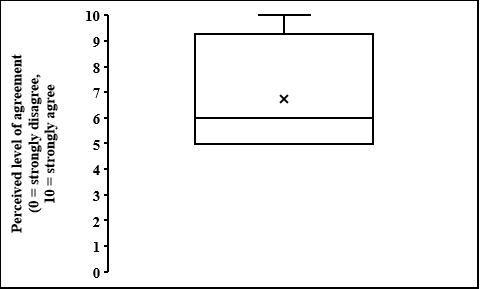


**Mean = 7**

**Median = 7**

**IQR = 5**

Panellists also suggested some useful additional comments for the existing statements which have been acknowledged by the research team. These comments will be included in the final write up for each statement. Furthermore, the research team are satisfied that all statements have achieved the desired outcomes for a consensus. We would like to thank all of our panellists for their patience, dedication and time for taking part in the study. The research team would like to present the finalised statements below based on responses from all three rounds. These statements will be included in the final write up of the study to be sent for publishing at a later date.

1. **Finalised statements**

The following sixteen (N = 16) statements achieved the desired consensus (≥70%, median score = ≥7/10) over the three rounds of questionnaires.

1. Reasons for the collection of maturity-related data include concerns about overuse growth related injuries and to identify players at immediate or future risk of injury.
2. Players with deficits in movement efficiency might demonstrate a greater risk of growth-related injuries, however more research is needed given the quality of current evidence.
3. It is difficult to predict which players with deficits in movement efficiency will go on to experience poorer long-term injury risk outcomes. This could be improved with better equipment and education.
4. Functional assessments that explore “adolescent awkwardness” seem a promising but under investigated approach. In principle, it may facilitate conversations with performance staff to help them understand the mechanisms by which deficits in movement competency around PHV increases injury risk and can subsequently influence on-pitch performance.
5. Maturity-related data allows performance staff to monitor and adjust training load especially for those players closer to PHV. However, it should be conducted in a way that considers the individual, their environmental context and any extra-curricular or school activities the individual may partake in.
6. Growth-related data can be complimented with performance-related data to identify both early and late maturing players and also to determine whether players need to play across younger or older chronological age groups.
7. Maturity-related data needs to be presented to coaches using a personalised approach based on their individual preferences, due to the consequences of data misinterpretation on player development, selection and training load management.
8. Medical scanning techniques can provide greater reliability, validity and sensitivity for maturity-related assessments, but are unlikely to be used in a real world setting due to ethical and financial implications.
9. Players who are before or during PHV, would benefit from an increased frequency of maturity and injury screening assessments from 12-week to 6-week intervals. This could help to closely monitor the physiological processes associated with an increased risk of injury, providing that measurements are taken accurately.
10. Longitudinal and standardised maturity-related data collection is preferable as it allows for a more accurate representation of maturation and its effects on injury risk over the course of the season(s), as well as identifying other inherent injury risk factors and players who are at an increased risk of injury.
11. Maturity-related risk factors with the highest consideration for injury prevention include accelerated growth rates, imbalances between muscular strength and flexibility, abnormal movement mechanics, the period during and after age at PHV, reductions in neuromuscular control and a players' maturity status (% predicted adult height).
12. Multidisciplinary approaches towards training/game load management, S&C interventions and consideration of injury history are the most effective strategies to limit the effect of maturity-related injury risk factors.
13. It is unrealistic for practitioners to use bio-banding as a method to reduce injury risk without greater training and research dissemination.
14. Additional training and education are required surrounding the prescription of interventions for academy players with growth-related conditions such as Severs disease or Osgood-Schlatter’s.
15. Qualified performance/sports science staff in academy environments have sufficient knowledge and expertise of taking growth-related measurements and using common maturity assessment methods in practice (e.g. Khamis-Roche, 1994; Mirwald, 2002) to determine a players’ maturity status and the timing of the adolescent growth spurt.
16. Apophysitis conditions around the hip are more difficult to diagnose than apophysitis conditions around the foot and ankle and require a specialist assessment.
