# Supplementary material for: Consensus on maturity-related injury risks and prevention in youth soccer: A Delphi study
Source: PLoS One. 2024 Nov 12;19(11):e0312568. doi: 10.1371/journal.pone.0312568 (PMC11556685; doi:10.1371/journal.pone.0312568)
Supplement: Attachment — Submitted filename: Response to reviewer.docx [file pone.0312568.s006.docx]

Dear Editor,

Thank you for completing a review of the manuscript entitled: Consensus on Maturity-Related Injury Risks and Prevention in Youth Soccer: A Delphi Study. (Manuscript ID: PONE-D-24-23052).

My co-authors and I appreciate your time and feedback in reviewing the manuscript and are pleased you appreciate the value of the research. In accordance with some of your recommendations we have made the following changes to the manuscript and provide a detailed description below of the changes we have made. Following these revisions to the manuscript we consider it now ready for publication.

Once again, thank you for your time and feedback. We look forward to hearing your decision.

Yours sincerely

The authorial team.

| **Reviewer feedback** | **Authorial team response** | **Revisions made to the manuscript** |
| --- | --- | --- |
| **1. “**Please ensure that your manuscript meets PLOS ONE's style requirements, including those for file naming**.”.** | The authors appreciate this comment and have made a conscious effort to adjust the formatting of the manuscript to adhere to PLOS ONE’s style requirements. | See manuscript. |
| **2. “**Please review your reference list to ensure that it is complete and correct**”.** | We acknowledge that some references needed adjusting and have updated all references to ensure they comply with the journals requirements. The authors can confirm that we used no retracted references during the write up of our research. | See references section. |
| **“**I have some concerns about the citations and statements in the article. I suggest authors to think twice while using the word “holistic development”. Throughout the paper, there are some places this phenomenon mentioned but I believe that it is not appropriate to do so. For example, in the sentence starting on Line 705 “This study highlights that maturity related data is collected and used holistically, to support the long-term development of players from physical, performance-related and psychosocial perspectives, but not for recruitment or retain/release decisions.”, here in this study I cannot see any concern or statements for psychosocial or emotional perspective even in participants’ notes provided as supplements.**”** | We would like to thank the reviewer for expressing their opinion on this topic. When we referred to the term ‘holistic development’, we were implying that academy systems attempt to develop their players in a variety of ways, that expands beyond physical/performance-related development. Therefore, we believed using the word holistic would be a suitable representation of our contention. We have removed the word holistic and have attempted to re-phrase this concept throughout the manuscript, in light of the reviewers’ comments. | See manuscript for general changes made throughout. |
| **“**Another one might be the sentence starting on Line 521: Firstly, maturity-related data collection is completed for multiple purposes, to support the long-term development of players from a holistic perspective (12). I think the paper cited here with number 12 does not have any connotation for collecting data with a holistic perspective either. So, I suggest authors to think twice on the phenomenon (i.e. holistic development/perspective) and do the revisions accordingly in their paper**”** | This study by Arenas et al., (2023) aimed to establish the main motivations and importance of maturity-related data collection using thematic analysis in German academies. We used the phrase ‘holistic perspective’ to demonstrate the variety of themes they proposed when completing maturity-related assessments with players. We have removed this phrase to say “Firstly, maturity-related data collection is completed for multiple purposes to assist with the long-term development of players”. | See manuscript- line 521 |
| **“**Moreover, for some citations throughout the paper, I suggest authors to go to the very first paper saying/showing/presenting/giving that specific finding or suggestions. For example, in the sentence starting on Line 70 “For example, youth players of the same chronological age group can vary in biological maturity by as much as 5-6 years (2).”, here the paper cited with number 2 does not have any data regarding 5-6 years difference. So, the very first source should be found and cited here which have this finding.**”** | We acknowledge this comment by the reviewer and have used the original source as the reference for this statement. This is now reference number 3. | See manuscript- line 70 |
| **“**Again, in the sentence starting on Line 111 “These findings suggest that the non-invasive methods used to assess maturity status and timing in youth players are largely unreliable, which could have negative consequences for players’ injury risk (2)”, the same cited paper number 2, does it say so? Does it say that the degree of non-invasive methods’ predictions on maturity status and timing has negative consequences for players’ injury risk? Does this article really suggest so? Are they “largely” unreliable?.**”** | The authors acknowledge they may have been speculative in making this statement, given the lack of evidence to prove a link between injury risk and the methods used to complete maturity-related assessments. We have published a review (Sullivan et al., 2023) that investigated the reliability of invasive/non-invasive methods used to complete maturity-related assessments in youth soccer players. However, this couldn’t make a definitive link between the type of method used and injury risk. Therefore, the statement has been adjusted, including the removal of the original reference and replacing it with a more suitable reference now number 10. | See line 111 |
| **“**Lastly, can authors check whether it is citation number 3 or number 1 suggesting the statement in the sentence starting on Line 71 “The variability in the timing of the adolescent growth spurt (occurring between 13-15 years in boys) can offer additional complexities for performance staff who wish to implement injury risk and training load management strategies (3, 4). So, again, I suggest authors to think twice for their citations**.”** | The authors have checked this reference and can confirm it was reference number 1 making this statement “the inter-individual variation in maturation during adolescence is large, making it difficult for practitioners to accommodate for differences in growth and maturational status”, this has been combined with Wik et al. (2024) who stated “An accelerated period of somatic growth is observed during the adolescent years, with peak height velocity (PHV) and peak weight velocity (PWV) for an average boy occurring around the age of 13 to 14 years and 14 to 15 years, respectively [4]. There is, however, a wide range in both timing (age at PHV from 12 to 17 years) and intensity (PHV from 5 to 12 cm/year)”. Collectively from these statements, we have concluded that there is large inter-individual variation in the timing of the growth spurt in players, and this large variation, between the ages of 13-15 years, increases the challenge and difficulty for practitioners to assign appropriate training loads and injury prevention strategies on a group basis. The statement has now been referenced appropriately to use reference 1 and not 3. | See line 71. |
| **“**I suggest authors to stress more and explain why they chose Delphi design because I really think that it is important for this study and makes the article stronger.**”** | We thank the reviewer for appreciating the study design and have used their comments to justify the use of a Delphi for the study. The updated justification can be found under the Delphi design subheading in the methods section. | See methods section- Delphi design subheading. |
