# Supplementary material for: Consensus on maturity-related injury risks and prevention in youth soccer: A Delphi study
Source: PLoS One. 2024 Nov 12;19(11):e0312568. doi: 10.1371/journal.pone.0312568 (PMC11556685; doi:10.1371/journal.pone.0312568)
Supplement: Attachment — Submitted filename: Response to reviewer.docx [file pone.0312568.s007.docx]

Dear Editor,

Thank you for completing a review of the manuscript entitled: Consensus on Maturity-Related Injury Risks and Prevention in Youth Soccer: A Delphi Study. (Manuscript ID: PONE-D-24-23052).

My co-authors and I appreciate your time and feedback in reviewing the manuscript for second time and are pleased you appreciate the value of the research. In accordance with your recommendation, we have adjusted the manuscript and provide a detailed description below of the changes we have made. Following this alteration to the manuscript we consider it now ready for publication.

Once again, thank you for your time and feedback. We look forward to hearing your decision.

Yours sincerely

The authorial team.

| **Reviewer feedback** | **Authorial team response** | **Revisions made to the manuscript** |
| --- | --- | --- |
| “In the revised document provided to me: You have cited “Johnson, A. Monitoring the Immature Athlete. Aspetar Sports Medicine Journal. 2015; 4(1)” for your statement “For example, youth players of the same chronological age group can vary in biological maturity by as much as 5-6 years”. My question again: Does this article have empirical hard data or is it written there with referencing to another article? The answer is: Johnson (2015) did not have it either and just cited another previous paper for this statement which is “Johnson, A., Doherty, P. J., & Freemont, A. (2009). Investigation of growth, development, and factors associated with injury in elite schoolboy footballers: prospective study.”….. “The original data comes from Malina et al.’s (2000) study related to that 5-6 years difference. So, please cite Malina et al.’s study instead of Johnson’s study for your statement because the hard data comes from Malina et al’s study. That would be ethical to giving their credits and dues considering the work they did.” | The authors thank the reviewer for correctly identifying the original source of the information to support the statement we have made in the manuscript. In light of this, we have changed our reference number 3 from Johnson et al., (2015) to Malina et al., (2000) as suggested and correctly pointed out by the reviewer. | See references section- Reference number 3. |
